# Soft-Tentacle Gripper for Pipe Crawling to Inspect Industrial Facilities Using UAVs

**DOI:** 10.3390/s21124142

**Published:** 2021-06-16

**Authors:** F. Javier Garcia Rubiales, Pablo Ramon Soria, Begoña C. Arrue, Anibal Ollero

**Affiliations:** GRVC Robotics Laboratory, University of Seville, Avenida de los Descubrimientos, S/N, 41092 Seville, Spain; fragarrub@alum.us.es (F.J.G.R.); prs@us.es (P.R.S.); aollero@us.es (A.O.)

**Keywords:** UAVs, inspection, soft robotics

## Abstract

This paper presents a crawling mechanism using a soft-tentacle gripper integrated into an unmanned aerial vehicle for pipe inspection in industrial environments. The objective was to allow the aerial robot to perch and crawl along the pipe, minimizing the energy consumption, and allowing to perform contact inspection. This paper introduces the design of the soft limbs of the gripper and also the internal mechanism that allows movement along pipes. Several tests have been carried out to ensure the grasping capability on the pipe and the performance and reliability of the developed system. This paper shows the complete development of the system using additive manufacturing techniques and includes the results of experiments performed in realistic environments.

## 1. Introduction

The use of unmanned aerial vehicles (UAVs) has grown exponentially during the last decade. This growth has been associated with technological improvements, such as those in navigation systems and perception sensors.

Nowadays, there is an increasing interest in the use of UAVs for inspection and maintenance. At the moment, most inspection and maintenance tasks are carried out manually which exposes the operators to many dangerous situations. This paper focuses on facilities where there are tons of tubes and pipes that are required to be inspected, as can be found in the oil and gas sector.

In oil and gas production plants, some components degrade. The excessive corrosion of pipelines can lead to accidents, catastrophic failures, impact the environment, and affect plant availability. To prevent this situation, inspection processes such as wall thickness measurements are performed to ensure that plants have safe operating condition, or provide alerts for corrective actions if needed. These activities manually performed by operators. The main problem is that the structures to be inspected are in elevated locations at high temperatures or with toxic materials. This comes at a considerable cost to ensure the safety of inspection personnel and production outages.

By using UAVs, the operators are capable of inspecting inaccessible or dangerous zones without facing any risk. Furthermore, embedding sensors and cameras on the UAV allows them to perform more complex inspections. However, these operations using UAVs are still performed by manual control. The future of these applications relies on current research into the automation of these aerial systems.

For the accurate contact inspection of pipes with drones, landing gear is beneficial because it allows static contact to enable the UAV to perform measurements by coupling to pipes without causing any damage. Moreover, we are proposing a system that should also allow the robot to crawl along the pipeline. The soft gripper that is proposed in this paper is capable of having the necessary strength to hold onto the pipes, and move along them without causing damage.

Then, in addition to saving energy, compared to UAVs that can only fly, our hybrid (flying and crawling) locomotion system presented in this paper does not require the ability to accurately land on the inspection point because it can crawl after landing to be positioned where desired. The proposed system also has other benefits, compared to conventional crawlers, since its flying capability allows it to access places which would pose challenges for a human operator.

The idea is to use soft materials for the landing gear attached to the UAV as this is a safer alternative than other methods used in the state of the art. The use of soft materials in this area is a novelty when integrated into aerial robots. The problem is the difficulty of designing a lightweight gripper that is at the same time compact, energy-efficient and reliable.

Soft materials increase the adaptability of the holding system, while ensuring lower damage to the structures. This is an ideal solution for typical pipe inspection tasks in industrial facilities.

The ultimate goal is to have a system capable of crawling through pipes and inspecting them with ultrasonic sensors and make non-destructive testing (NDT) inspections. This kind of solution is very interesting for the industry and related service providers, as they can save costs, time and prevent undesirable accidents.

The rest of this paper is divided into five parts. The second section reviews the previous work. The third section describes the soft landing gear system, including the design, manufacturing process, and the operation flow of the landing gear and the soft limbs. The fourth section discusses the validation of the proposed system and the experiments carried out to validate its functioning. In the fifth section, the flight tests with the final setup is described and evaluated. Finally, our conclusions are drawn in the sixth section.

## 2. Related Work

Soft devices are currently being used in many areas of robotics because they provide advantages that the more traditional systems do not have, such as adaptability, compliance, better interaction with the environment and multi-functional end-effectors. Some examples of bio-inspired systems that can be used to interact with people are lightweight compliant arms with soft muscles that are pneumatically activated [1] and a pneumatic actuator can also be used to try to imitate the movements of a fish [2]. There are also soft grippers with muscles that are pneumatically activated to work in industrial environments interacting with humans [3].

There are many examples of materials and technologies, such as dielectric elastomer actuator (DEA), silicone-based elastomers, 3D-printed flexible actuators, or pneumatic actuators [4,5,6,7]. The main limitations associated with these soft-based actuators tend to be related to the complexity of the manufacturing process.

Other authors’ examples are the high-contraction ratio pneumatic artificial muscle (HCRPAM) [8,9], prosthesis and grippers for manipulation [10], robots with elastomer actuators [11] and horticultural manipulation applications [12]. However, the main disadvantages of these actuators are the weight and space required by the pneumatic systems, which have motors and compressors with relatively high dimensions and weight.

Soft robotics are starting to be used in UAVs aiming to develop systems with capabilities to manipulate delicate objects, and to interact with people while flying. The use of soft materials is explored in [13] to become collision-resilient and increase its robustness. A special folding mechanism was investigated in [14]. DEA artificial muscle has also been used to try to simulate flapping wings, insects [15], or using a flexible membrane based on origami folding to preserve structural integrity during collisions [16]

The soft and compliant nature of the actuators ensures that soft robots are able to provide a safe interaction between the system and the facility to be inspected. Despite being lightweight structures, they are capable of achieving a high degree of freedom and a high force-to-weight ratio.

A large variety of robots have been designed to inspect pipes internally [17,18]. In fact, the most popular method of inspection is intelligent pigging [19,20], which makes use of devices equipped with sensors that navigate inside the pipes carried by the pipeline’s fluid.

Robots have also been used to inspect pipes externally, usually called crawlers [21]. These robots commonly use magnetic wheels or tracks to move along the surface of pipes. This is very useful because the crawler can go underneath the pipe to make measurements, detecting possible leaks or corrosion. They are able to move freely over smooth surfaces, but in general, they cannot overcome obstacles and are limited to magnetic metal pipes.

The main locomotion alternative to these magnetic crawlers consists of an annular structure equipped with wheels, which obtain their adherence from a vacuum sucker.

UAVs can reach inaccessible areas faster than human operators or crawler robots. However, their flight endurance is very limited, and these robots are mainly used nowadays to perform visual inspections on structures by using different types of cameras (color, stereo, infrared), lasers, or other sensors. Authors in [22,23,24] proposed the use of UAVs to detect gas leaks and to monitor and map pipes. However, these solutions only allow for the visualization of surface damages.

Most recent research focused on the development of aerial robots that are able to not only perceive but also interact with the environment. This can be achieved by using robotic arms with several degrees of freedom (DOFs) that are attached to the UAV to interact and perform contact inspections [25,26]. These robots enable a new kind of application in which robots will be able to not only inspect but also perform the maintenance tasks at the industrial facilities [27]. These types of systems are called aerial robotic manipulators or aerial manipulators (AMs).

As related with previous perching mechanisms for UAVs, in [28], a single soft gripper was embedded at the bottom of a UAV to perch on pipes for inspection and maintenance tasks. Similar approaches have been taken in the rigid landing gripper of [29], the semi-soft perching system developed in [30] and the bio-inspired UAV with a soft landing gear that [31] used to land. However, these designs did not tackle the problem of moving along the pipe. The system presented in this paper allows crawling over the pipes to inspect them, saving time of flight.

## 3. Soft Landgear

In this section, the design of the landing gear and its characteristics are described. The section is split into two parts: the first one focuses on the description of forward-motion mechanism, while the second one focuses on the design and mechanical properties of the soft limbs.

**Figure 1:**
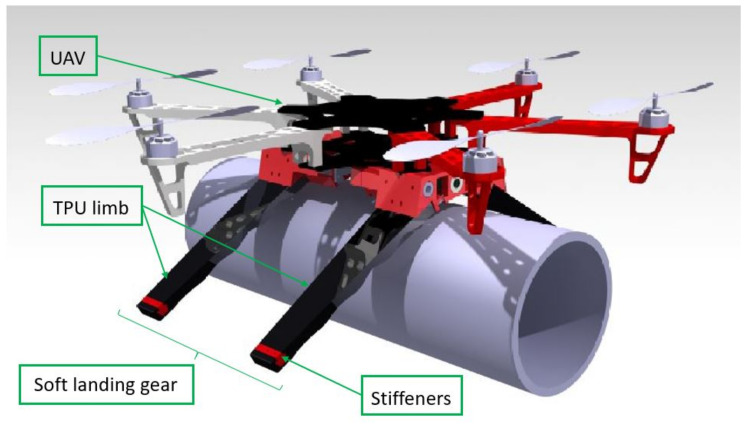
CAD design of the complete system.

The complete mechanism gear has been designed to allow the robot to crawl over the pipe. A compact and functional design that can be used for a variety of pipes because of the flexibility of the limbs is shown. Figure 1 shows the complete CAD design.

The gripper is manufactured with three different materials, which are: TPU, PLA and ecoflex. TPU (thermoplastic polyurethane) is a linear elastomeric polymer that can be used for 3D printing. Its greatest qualities are the flexibility and durability of the material. Polylactic acid (PLA) is a common plastic material in 3D printing. Finally, ecoflex is a cure silicone rubber compound. The advantages of ecoflex are that it mixes its components in equal parts to obtain a smooth and moldable silicone that can gain any shape and greatly increases the adhesion.

### 3.1. Forward-Motion Mechanism

The forward-motion mechanism is a rigid core designed to be manufactured in PLA (as mentioned earlier). Additionally, all of the electronics are placed in this part because the rigid casing is more robust to possible impacts.

It has been designed to be flat on the upper side to make it easier to attach to different drones. This attachment is done with an embedded electromagnet. This special device is able to switch the magnetic field so that it can be enabled or disabled with a pulse. This can be used, for example, in situations where gas is detected or in emergency cases. This functionality is further explained in our previous work [32].

The mechanism is composed of two pieces: a fixed part that is attached to the drone and a mobile part that generates the forward movement. These two parts are symmetrical. The objective is to make a compact and robust design with the lowest possible weight so that it can be easily transported by the UAV, while the center of gravity does not significantly change when the UAV is moving.

**Figure 2:**
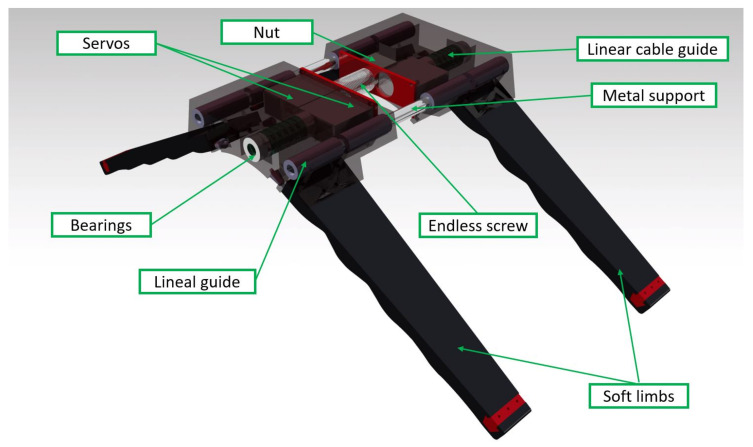
CAD view of the forward-motion system. The arrangement of the three servos, the worm gear, the linear bearings at the ends of the case and the guides to contracting the soft limbs can been seen.

Figure 2 shows the configuration of all the components. Three servomotors are responsible for all of the movements. Two of the servos are used to fold the soft limbs, which will be described later in Section 3.2. The other servo actuates as an endless screw that produces the forward movement. To restrict the torsion of the endless screw, two linear guides are located at the extremes of the rigid parts, which are attached with two 8 mm bars.

The lower part of the landing gear is a half-cylindrical section for the better adaptation to the pipes where it lands. The reference pipe size for this circular section is 160 mm, but the system can work in a diameter pipe range between 100 and 300 mm. On the sides, the system has two flaps protruding from the structure to attach the soft limbs.

### 3.2. Soft Limb Design

This section presents the design of the soft limbs. The points of interest of the soft-tentacle gripper are its capabilities of adapting to pipes of any diameter and absorbing the impact on the pipe while landing.

Each limb is made out of TPU. This rubber-like material provides the gripper with enough rigidity to retain its shape and maintain the exerting forces, but also enough elasticity to bend and adapt to different pipe shapes.

Another benefit of this material is that it can be used by a 3D printer, making it possible to easily iterate and develop different limb shapes. Finally, the tip of the limb is coated with silicone to increase the grip force of the system and obtain a softer contact with the pipe.

The main property of the gripper is that it is intrinsically compliant, allowing it to easily adapt to variations on the diameter of the pipe. Furthermore, weight is a crucial variable in aerial systems, where any additional payload means less flight time and it also affects the maneuverability of the UAV. Therefore, the landing system to be 3D printed has been designed to keep the weight to a minimum. Finally, the soft approach also offers more safety in the case of crashes.

**Figure 3:**
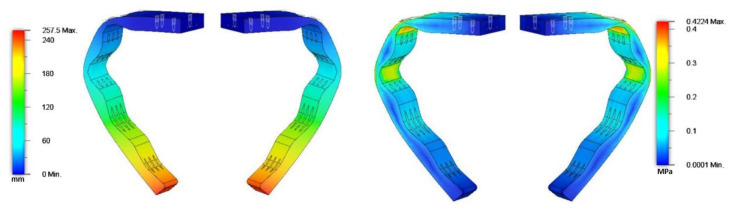
The left-hand image shows a study of the deformation for non-linear materials and the right-hand image shows a study of the stress.

The selection of the shape of the limbs has been decided after various tests and simulations, by observing the limb’s deformation and finding the best adaptive shape for the pipe. In the studies carried out, the properties of the TPU material were analyzed. An example of simulation for the deformation of the limb is shown in Figure 3.

**Figure 4:**
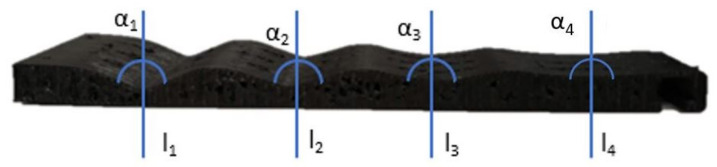
The image shows the final impression of the limb and the different angles chosen in its design.

To obtain a good grip and have the highest diameter range, the stiffness of the joint had to be taken into account. The stiffness formula for each joint is $j_{i}=EI/L$, where E and I are two constant parameters: E is the Young’s modulus (the one used for the TPU is 100 N/mm${}^{2}$), and *I* is the cross-sectional moment of inertia. L is the length between the nylon thread and the flexible segment. Figure 4 shows the final shape of the limb.

**Figure 5:**
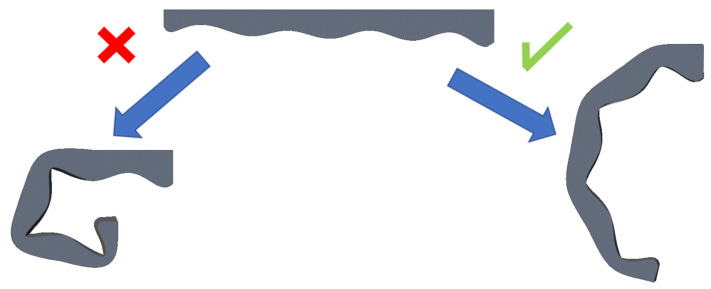
Example of the bad fold of soft limb and good fold when changing $j_{i}$ parameters.

The first tests were made with $j_{1}=j_{2}=j_{3}=j_{4}$, where $j_{1}$ is the closest joint to the UAV and $j_{4}$ is the joint that is farthest away. This means that all lengths (L) were equal and, therefore, the same stiffness was generated at each joint of the soft limb. With this configuration, the limb started to bend first on the tip, which implied that it was not adjusting properly to the pipe, as shown in the left-hand example of Figure 5.

After this first attempt, the lengths $L_{i}$ were changed, making them higher in the joints that are closer to the UAV, and reducing it progressively in the joints next to the tip; i.e., $j_{1}<j_{2}<j_{3}<j_{4}$. With this approach, the tentacles bend better toward the shape of the pipe, increasing the grip of the system. The resulting stiffness generates the fold pattern that is shown in the right-hand example of Figure 5.

The following equation describes the recursive formula for the joint’s stiffness [33]. The Equations in (1) indicate the curvature generated by the limb joints:

(1) $$\begin{array}{c} j_{n}=\frac{1}{\theta_{n}}(F_{t}(m_{n}g\left(l_{cn}\cos\sum_{n=1}^{N}\theta_{n}-\frac{h}{2}\sin\sum_{n=1}^{N}\theta_{n}\right)\\ +l_{\alpha n}\sin\left(\frac{\theta_{n}}{2}\right)+h))\\ R_{xn}=F_{t}-m_{n}g\sin\sum_{n=1}^{N}\theta_{n}\\ R_{yn}=F_{t}\frac{\theta_{n}}{2}-m_{n}g\cos\sum_{n=1}^{N}\theta_{n} \end{array}$$

**Figure 6:**
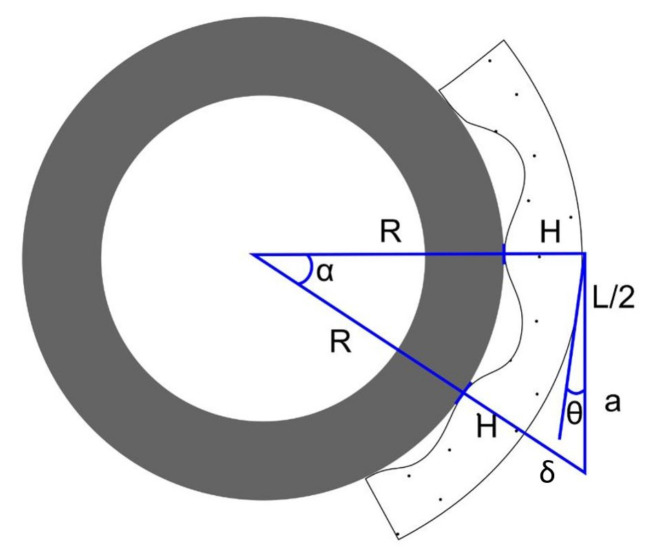
Model used to determine limb deflection in a pipe. H is the height of the limb, L is the length of the link, R is the radius of the pipe, θ is the deflection of the joint, and α is the angle between the contact points of two consecutive links.

These equations have been used to determine the joint deflections required to grip a circular cross-section of a particular radius R formed with θi=α angles. Figure 6 shows an example of how the limbs adapt to the circular section of a pipe, showing the different parameters used for the calculation. This design radius is considered to be optimal. Nevertheless, due to the softness of the framework, the gripper can envelope pipes of larger and smaller radius. Equations can be used to solve θi, giving as a result:

(2) $$\left\{\begin{array}{c} cos\left(\alpha \right)=\frac{R+H}{R+H+\delta }\\ tan\left(\alpha \right)=\frac{\alpha +L_{i-1}/2}{R+H}\\ cos\left(\theta_{i}\right)=\frac{L_{i}}{2a}\\ tan\left(\theta_{i}\right)=\frac{2\delta }{L_{i}} \end{array}\right.$$

(3) $$\theta_{i}=\tan^{-1}\left(\frac{2\left(L_{i}^{2}+L_{i-1}+L_{i}\right)\left(R+H\right)}{L_{i}\left(4{\left(R+H\right)}^{2}-L_{i}^{2}\right)}\right)$$

By solving Equation (2), the system obtains δ and substitutes δ into $tan(\theta_{i})$ and Equation (3) is obtained.

Finally, by knowing each $\theta_{i}=\alpha$ angles for each joint, we can obtain $\beta_{i}$, the bending angle, from the following equations:

(4) $$\left\{\begin{array}{c} S=tan(\alpha )R\\ \beta_{i}+2\psi_{i}=180\\ cos\left(\psi_{i}\right)=\frac{S/2}{Z}\\ sin\left(\beta_{i}/2\right)=\frac{S/2}{Z} \end{array}\right.$$

**Figure 7:**
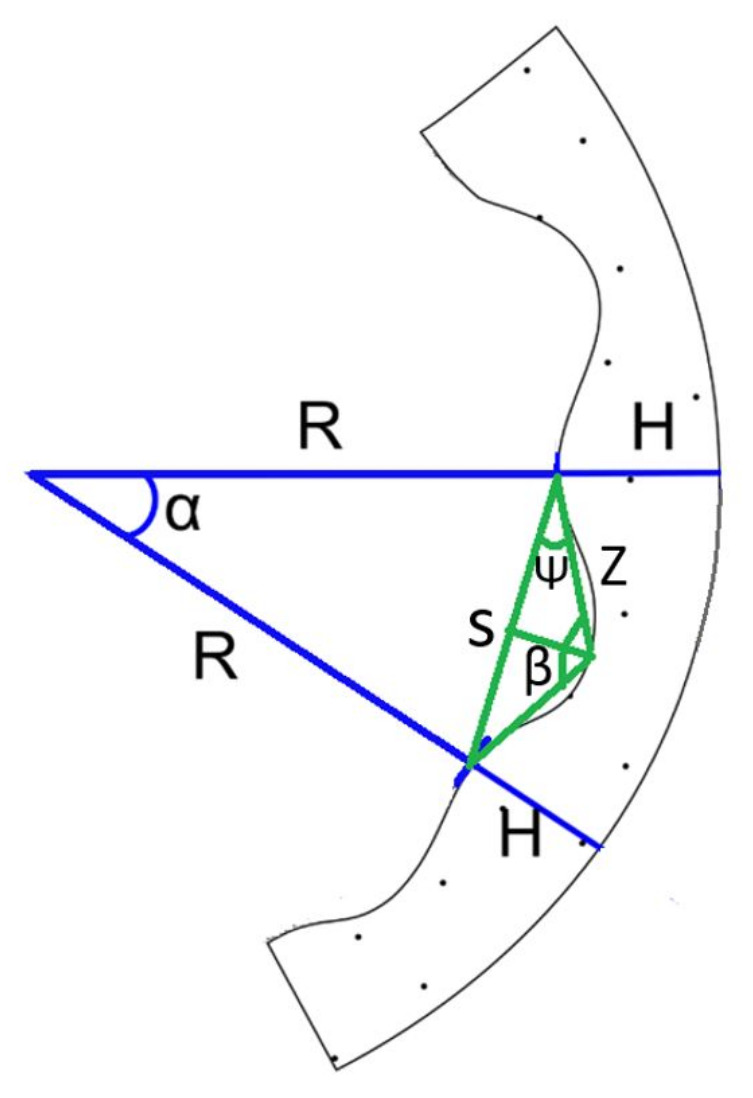
Model for the determinate used to determinate the β angle.

Figure 7 shows all the parameters used to solve the system of Equation (4):

The conclusion was that to obtain the best fit possible of the limbs to the pipe, the shape of the limbs should suffer a progressive deformation from the base to the end of the limb. Table 1 shows the selected angles obtained for the selected radius R, the tendon tension $F_{t}$ and the dimensions of the limbs.

### 3.3. Complete Locomotion System

This subsection explains that all of the components conform to the locomotion system, and how they work. The locomotion system consists of three servomotors, which are embedded into the frame of the soft landing gear (as explained earlier).

**Figure 8:**
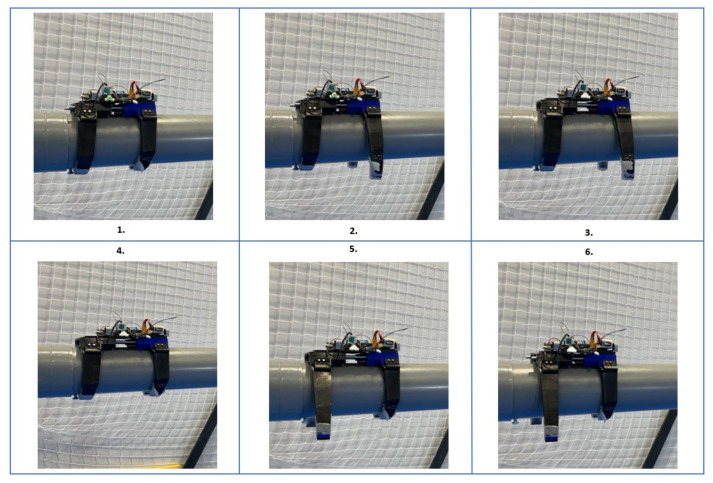
Example of movement sequence of the soft landing gear: (in **stage one**), it grips to the pipe; (in **stage two**), it opens the front limbs (blue case); (in **stage three**), it moves forward; (in **stage four**), it closes the front limb (blue case); (in **stage five**), it opens the rear limbs (black case); and finally, (in **stage six**), it moves the rear part.

Two of the servomotors are used to bend the soft limbs. The third is used to perform the linear displacement of the landing gear, which is made with an endless screw. Figure 8 shows the sequence of movements made by the soft land gear.

The first step to select the servomotors is to calculate the required minimum torque. The complete system weighs 3.25 kg, thus each limb will must exert ∼0.81 kg at its tip. Each motor has two limbs. For that reason, the final servo should exert at least 1.625 kg. By making this assumption, we grant that the gripper can hold the complete weight of the UAV in the worst scenario. Nevertheless, in most situations, this required strength will be lower, as part of the weight is held by the pipe, and the gripper only needs to prevent the UAV slipping laterally.

As shown in Figure 8, the motion runs as follows: two servomotors are hooked in pairs with the soft limbs using nylon threads, which allows them to open and close the limbs depending on the direction of the rotation of the motor. The third servomotor is responsible for the forward movement, using an endless screw that is connected to the motor at one end and to a nut at the opposite end. When the motor turns in one direction, the soft landing gear moves.

### 3.4. Soft Limb Manufacturing Process and Assembly

This subsection will describe how we manufactured the limbs that are installed on the landing gear. Once the design has been carried out and has fulfilled the specifications, the manufacturing process of the limbs begins. One of the most important challenges is to be able to manufacture a soft part in a 3D printer, while making it easy to replicate and ensuring that the process is accurate.

All of the designs are made with TPU and a pair of PLA stiffeners, all produced on a 3D printer. These limbs are printed with different infill and printing patterns. The limbs were tested in the complete setup, until the one with more flexibility was selected. It was tested with a 10 percent infill which was very flexible and did not maintain the desired curvature when it was pulled by the nylon, then it was tested with a 20 percent infill which was very stiff and hardly allowed the limb to bend when it was in tension. The limb with an infill of 15 percent and a square printing patron was chosen as the optimal case.

Once the process of the impression of the TPU limb was finished, a PLA stiffener must be added to the tip to ensure that the tip does not deform. A stiffener is used to prevent losing strength at the tip when the nylon threads are contracted. After this, the different nylon strands that exert the force to deform and obtain the circular shape of the pipe, should be added to the limbs. These nylon threads are attached to the servomotor of the corresponding locomotion system and is then hooked at the end of the limb with the help of the PLA stiffeners at the end of the limb.

**Figure 9:**
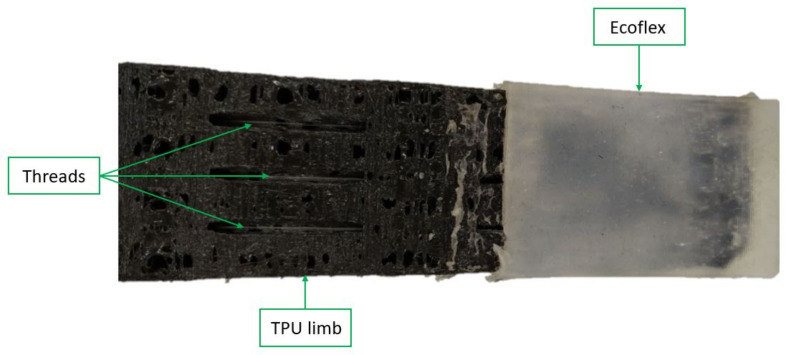
The final result when joining TPU with ecoflex. It can be seen that ecoflex is only applied to the tip to increase the adhesion in this area.

Finally, ecoflex is added. This elastomer is very flexible and rough. Ecoflex is applied to the tips of the limbs to improve adhesion to the pipe. To incorporate ecoflex with the TPU, molds were created using PLA with the shape of the limb tip and then were filled with ecoflex. The soft limb was then introduced into the molds, obtaining the silicone on the TPU. The ecoflex was cured for eight hours to obtain its physical qualities, and after this time, it was demolded. Figure 9 shows the final result of the operation.

With all the parts manufactured, everything is assembled as follows. There are two parts. The first is the fixed part, where the two motors are located. One motor is responsible for closing and opening the limbs and the second motor operates the worm screw. This part has two holes at the top where the couplers are inserted. These are joined with metal bars. The second is the mobile part, which incorporates in its interior only a motor to drive the limbs. It also contains some superior holes where the linear bearings are inserted so that this part can be moved through the metallic bars. It also contains a nut in the frontal part where the screw without end will be connected.

The last step of the construction of the soft landing gear is to join these two parts with the worm screw and the metal bars. The metal bars are added to give consistency and to carry the weight of the landing gear so that the worm screw is not exposed to too much stress. It should be added that the motors in charge of opening and closing the limbs have a reel that is fixed to it and a bearing. The reel takes care of rolling up the nylon threads, and these threads pass along the limb and stay attached to the stiffeners.

## 4. Soft Land Gear Validation

This section presents the validation tests of the mechanical behavior of the soft landing gear. Experiments have been carried out to measure the force exerted by the limbs using different pipe sizes. The deformation when the limbs close over the pipe and the maximum slope ranges that the system can withstand without falling (laterally) are also studied.

It should be noted that the results in this section demonstrate the reliability of the design, as well as the actual capabilities of the gripper. These results can be extrapolated for manufacturing other customized landing gears for different pipe sizes and other payload requirements.

### 4.1. Pull Force

In this section, a comparison is made between various servos, checking the force that can be exerted both experimentally and theoretically. Then, one must choose the one that meets the design expectations and has the least weight and dimensions.

At first, the grip force is evaluated via experiments closing the claw on a test bench and measuring the force with a dynamometer. The dynamometer is hooked to the claws and pulled upwards to give real force exerted by the servomotor.

For those tests, the base of the limb was fixed and a force was applied at the tip. This force is equal to that exerted by the nylon on the limb.

The servomotor voltage that is given as an example of the datasheet is validated with experiments, comparing the force output for a dynamometer applying this voltage with the theoretical force obtained in the datasheet.

A comparison has been made with three servos, the Feetech FS5103B, the Feetech SCS15 and the Feetech FT6325M. According to the technical specifications, the first servo has a voltage operation ranging from 4.8 to 6, the second has a voltage operation ranging from 6 to 8.4 and the third also has a voltage operation ranging from 6 to 8.4, whilst the force range obtained for each servo is as follows: for the first, the force ranges from 0.5 to 0.7 kg; for the second, the force ranges from 2.2 to 2.9 kg; and for the third, the force ranges from 2.8 to 3.6 kg.

**Figure 10:**
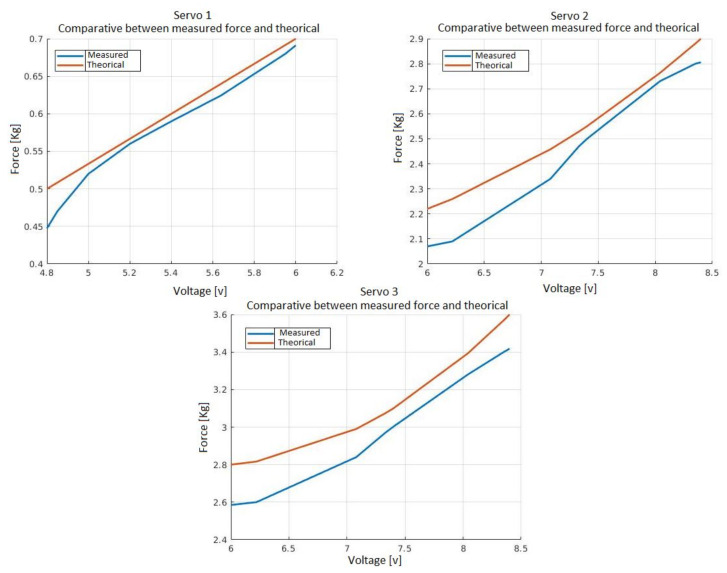
Comparison between the theoretical force and the real force performed by the soft limbs according to the voltage applied to the servos. The orange line represents the theoretical measurements and the blue line represents the experimental measurements obtained.

Figure 10 shows that the theoretical force is greater than the experimental force, which was expected, due to the normal mechanical losses. This loss is lower than the 4%, being bigger with lower voltages and lower with higher voltages. It is observed that the servo 1 graph does not perform the specifications while the second and third graphs do, and the third one more than complies with the specifications. Finally, the second one is chosen as it meets the design requirements and the servo has a lower weight.

After analyzing this information, we chose to use the Feetech SCS15 servomotors because they have enough force to move the landing gear and hold on to the pipes. In addition, they have a serial bus connection in which the three motors can be connected at the same time with the same bus and each motor can also be selected according to the motor ID. In Section 4.1, we will explain why this servo was selected.

### 4.2. Contact Pressure

This section introduces the experiments for measuring the pressure exerted by the soft landing gear on two pipes of 140 mm and 160 mm diameter. In these experiments, force-sensing resistors (FRS sensors) have been distributed all over the soft surface. These sensors have a resistance that changes when a force is applied to it. This measurement can be mapped to forces and extrapolated to pressure over the surface. In these experiments, we tried to understand the behavior of the soft limbs and the pressure areas, where the soft train exerts less pressure on the pipes, which exert more pressure. Several experiments were carried out to calculate the pressure of each limb. Once the experiments were carried out, the data were collected and averaged to later be processed and obtain the pressure map. This process was done for both 160 mm and 140 mm pipes.

**Figure 11:**
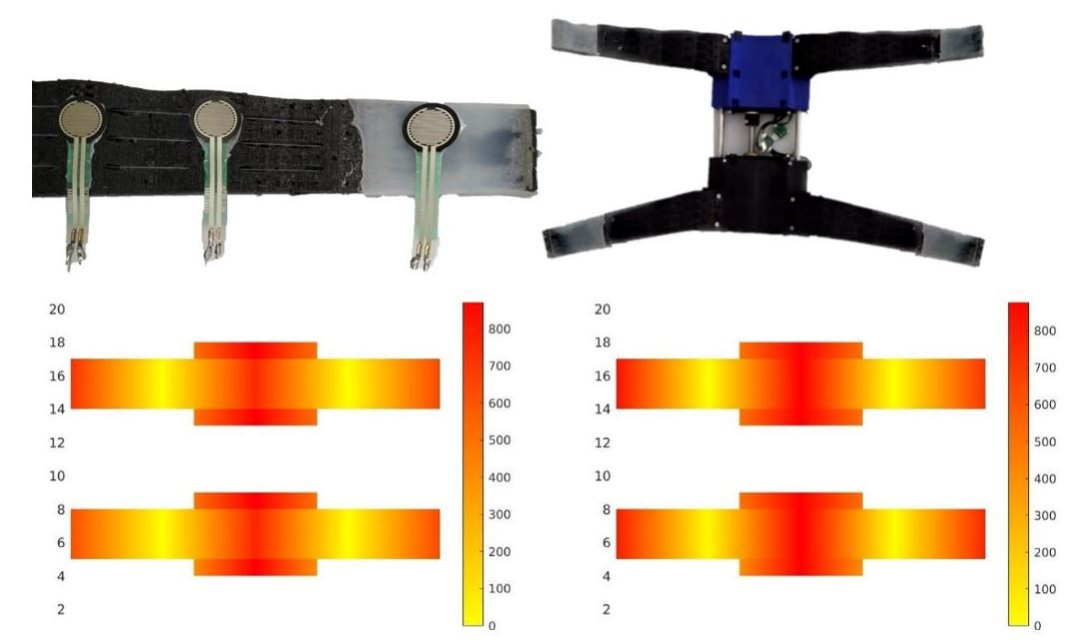
The upper image shows the lower part of the landing gear to which the pressure study is made. The lower left-hand image shows the pressure map made on a 140 mm-diameter pipe and the lower right-hand image shows the pressure map made on a 160 mm-diameter pipe.

Figure 11 shows that more pressure is exerted in the base of the soft train and also in the tips of the limbs. The areas where less pressure is exerted are the intermediate areas due to formed folds.

The same behavior is observed in both studied cases, in the pressure graph made on the 160 mm-diameter pipe and in the 140 mm-diameter pipe. The difference between these two graphs is that the general pressure recorded on the 140 mm pipe pressure graph is lower; that is, in general, there is less pressure at the end of the extremities and at the base of the soft train than on the 160 mm pipe.

### 4.3. Maximum Lateral Angle

**Figure 12:**
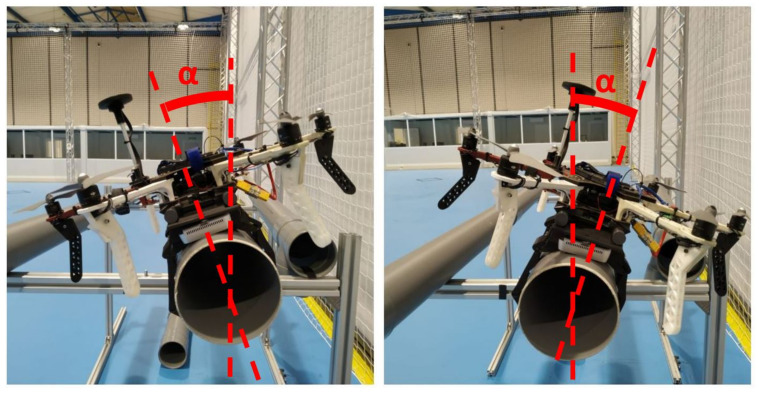
The maximum angle at which the landing gear can be grabbed with the drone on the pipe.

A study was also conducted to determine the range of the angle at which the soft train can be attached to the pipe without separating from it. For this test, a test-bench was installed in which a smooth PVC pipe of 160 mm diameter was placed and the maximum inclination angle concerning the vertical of the pipe was checked. The soft train together with the UAV was able to hold on to it with a maximum angle of 30 ${}^{{}^{\circ}}$C. Figure 12 shows the maximum angle at which the soft gripper can hold the contact to the pipe.

This experiment also tested the movement of the soft train and verified that the prominences arranged at the base of the train can pass over the pipe joints and their irregularities.

### 4.4. Crawling Gait Analysis

Finally, to analyze the repetitiveness of the movement of the system, a gait analysis using a motion capture (MoCap) system was carried out.

**Figure 13:**
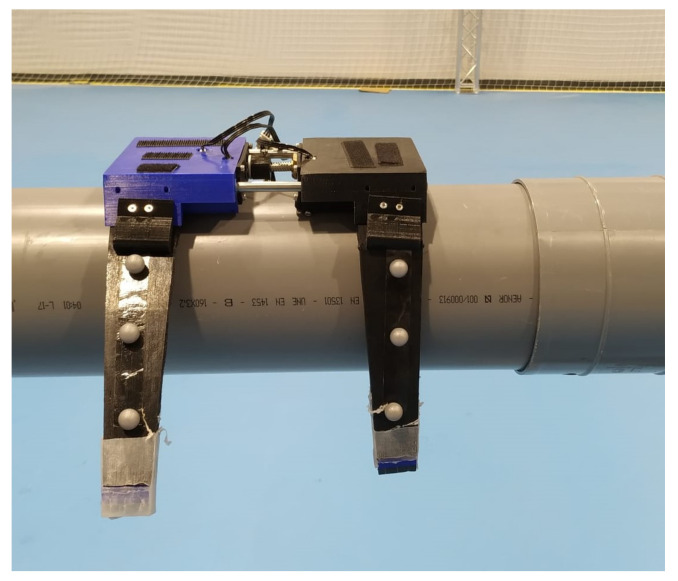
Placement of markers on a pair of soft limbs.

The MoCap system allowed us to record in real time the position of markers in a controlled movement. Three reflective markers have been placed at each limb, which are located at each joint where the limb is bent in Figure 13.

Knowledge of the position of the markers can be used to validate the motion on the pipeline. When the limbs are extended, the markers line up. As the limbs begin to bend, the markers move inward, forming a semicircle. Then, the displacement begins. First, the forward pair of limbs open and move forward. In the next step, the opposite happens: the front limbs are closed and the rear limbs open, which moves the mechanism forward. The motion must be linear on the pipe. Slippage is corrected by changing the center of gravity of the UAV by making adjustments when joining the soft landing gear to the main UAV platform to ensure the position of the center of gravity.

The recording of the position of the markers and the time can also be used to obtain the speed of the landing gear along the pipe. Thus, it has been obtained that the average speed is 4 cm/s.

Moreover, the position of the three markers on the limbs can also be used to obtain the radius of the circumference when closing the limbs. For the case studied, the radius is 84 mm.

Finally, it can be concluded that the movement made by the soft landing gear on the pipe is always the same, obtaining the same circumference radius. This also allows us to correct the center of gravity to prevent slipping, and to ensure that the UAV and the soft landing gear are centered on the pipe.

**Figure 14:**
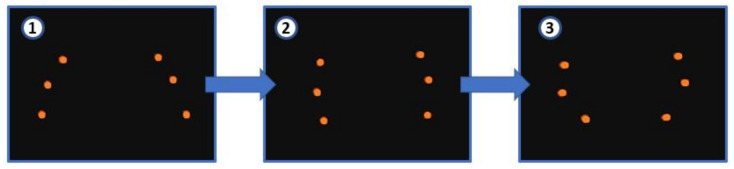
Closing sequence on the pipe and the limbs’ deformation.In the first picture, the tentacles are open. In the second, they begin to close. Finally, in the third picture, the limbs are completely close, adapting to the shape of the pipe.

Figure 14 illustrates the motion of the marker points in two limbs.

## 5. Experimental Test

This section describes the experimental setup of the whole working system, including the flying platform.

The soft landing gear was validated on a DJI FlameWhell 550 multirotor platform. This platform was chosen due to its versatile design. One of the advantages of the developed landing gear system is that it can be adapted to any type of multi-rotor system thanks to its modular design. It can also land on pipes of different diameters, and once landed, it can crawl along the pipe to perform both visual and contact inspection.

The motor controller board is in charge of supplying the necessary power for the motors to work and to control the motor sending the information through the data bus. An AVR-based board is used to control the landing and crawling on the pipe through the controller board, which executes the program that sequentially opens and closes the soft limbs to generate the movement of the system.

The AVR-based board is connected to an Nvidia tx2 onboard computer, which is in charge of sending the order received from the pilot and execute the high-level behavior software. However, this point is not the subject of this paper. It can also be used to integrate more sensors, including cameras. All these components are powered through specific voltage converters that regulate the power of each device from the battery.

**Figure 15:**
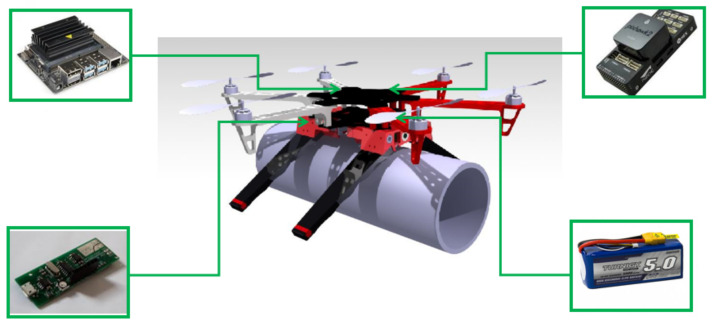
Setup including in the flying platform.

The AVR-based board sends the information by serial communication to the motor’s controller. This asynchronous Rx–Tx protocol has been used because it allows us to have two lines: one for transmitting to the motors and the other for receiving data such as the position, the speed, or which motor is working in each step. Figure 15 shows all of the components that we have used.

This UAV has two operation modes: manual and autonomous. In the manual mode, an operator sends the commands to move through the pipe. These commands are received by the Nvidia tx2 and transmitted to the AVR-based board via USB. The operator can select between either opening and closing the limbs, or either moving the soft train forward or backward. In the autonomous mode, the AVR-based board sends a sequential program to the motors so that they carry out a movement. A linear sequence of opening front limbs, moving forward, closing front limbs, opening rear limbs and moving forward is performed. This mode should be activated once the UAV is attached to the pipe.

**Figure 16:**
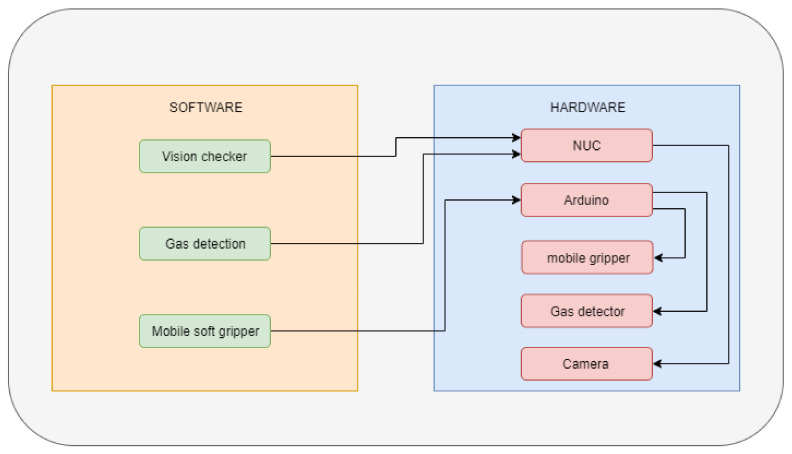
System scheme used.

Tests were carried out to verify the functionality of this unit. The first experiments that were performed on the pipe were the ones in which the soft landing gear moves through it. Once this stage was tested, the landing gear was installed on the multi-rotor system, in this case a DJI f550 which has a Pixhawk 2 for the control of the multi-rotor, an Nvidia tx2 as an onboard computer, a camera connected to it to locate and position the UAV on the pipe, and a gas sensor which is a safety sensor to detect a gas leak and preventing putting the installation at risk—which is further detailed in [32]. Figure 16 shows the scheme of the proposed system.

**Figure 17:**
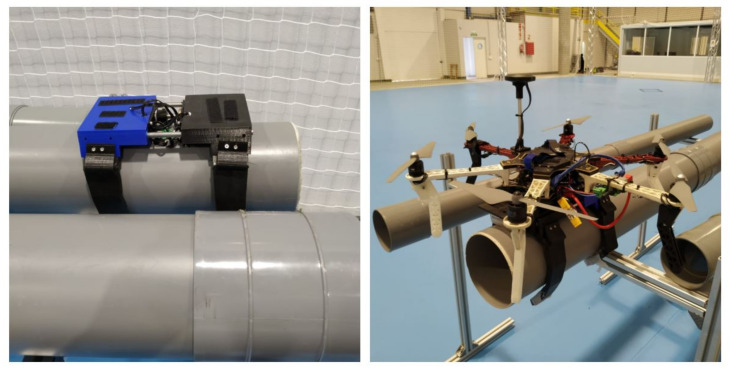
The image on the left-hand side shows the first experiment performed with the landing gear alone on the pipe. The picture on the right-hand side shows the complete system and how it is attached to the pipe.

Multiple experiments have been carried out to demonstrate that the system works under different conditions, including indoor and outdoor experiments with wind, which makes it more difficult to maneuver and land the UAV. The soft landing gear grips firmly to the pipe and helps center the multi-rotor to the pipe while landing. The grip is fast and safe and does not damage or scratch the pipe at any time, though the landing gear is strong enough to hold on to the pipe and move along it without needing to be stabilized with the UAV propellers. Figure 17 gives an example of the soft train attached to a pipe with and without the multi-rotor UAV.

The gripper system worked in all of the tests, demonstrating its reliability and ability to overcome joints between pipes while moving. It can also be attached to irregular surfaces.

**Figure 18:**
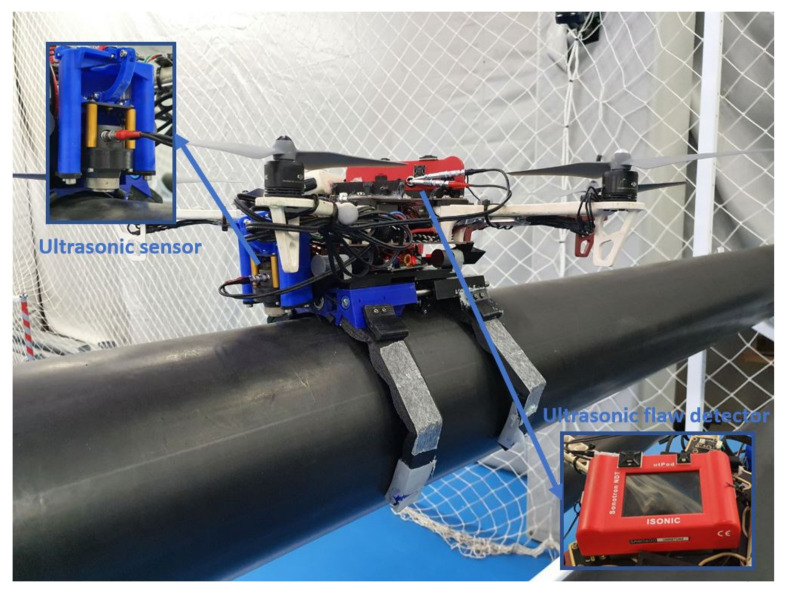
The image shows the complete system with an ultrasonic sensor and the flaw detection computer.

As proof of concept, an ultrasonic sensor was attached to the soft landing gear to acquire non-destructive information about the thickness of a typical steal pipe. Figure 18 shows the assembly and the components used for the inspection. The ultrasonic sensor is placed at the front of the landing gear and has a motor to move the sensor up and down. It is important to make the right pressure so it can take the measurements.

## 6. Conclusions

A novel design of a soft-tentacle gripper for UAVs to crawl on pipes was presented.

How the soft gripper was manufactured with 3D printing and molding techniques for the silicone-based material was also described. This manufacturing process is highly repeatable and reliable.

The design of the UAV landing gear is unique and is capable of crawling on pipes without damaging them. It also provides a fast coupling and decoupling of the pipe. It can move through welds and joints, without losing adhesion and without jamming. The soft mechanism also adapts to pipes of different diameters or even to non-cylindrical pipes.

The soft gripper was designed to be as lightweight as possible, easy to transport and easy to change. The system also has low-cost implementation and is easy to reproduce using additive manufacturing techniques.

The system has been validated with real tests landing and crawling on the pipes with an automatic system.

This system can be combined with various sensors to perform inspections, an example of which is the use of an ultrasonic sensor. This type of sensor combined with this soft landing gear is very useful for inspecting pipes located at high altitudes. This saves in terms of inspection costs and time and increases the safety of workers.

This research paper represents the first step to create a fully autonomous hybrid flying and crawling contact inspection robot that is able to operate in an environment with many obstacles.

Future work will focus on the development of a faster locomotion system with more degrees of freedom. Furthermore, a standard ultrasonic transducer will be introduced in the base of the crawl system in order to realize flaw detection over the pipe and to detect anomalies.

## Figures and Tables

**Table 1:** Final dimensions of the limb shape profiles.

Angle	$\beta_{1}$	$\beta_{2}$	$\beta_{3}$	$\beta_{4}$
degrees	132	136	168	175
length	l${}_{1}$	l${}_{2}$	l${}_{3}$	l${}_{4}$
millimeters	14	16	90	180

## Data Availability

Not applicable.

## Abbreviations

The following abbreviations are used in this manuscript:

UAV	Unmanned aerial vehicle
DOF	Degrees of freedom
DEA	Dielectric elastomer actuators
AM	Aerial manipulators
CAD	Computer-aided design
TPU	Thermoplastic polyurethane
PLA	Polylactic acid
ABS	Acrylonitrile butadiene styrene
EMF	Electromotive force
MoCap	Motion capture
NDT	Non-destructive testing
